# A systematic review of the impact of housing on sow welfare during post-weaning and early pregnancy periods

**DOI:** 10.3389/fvets.2022.903822

**Published:** 2022-08-23

**Authors:** Jen-Yun Chou, Thomas D. Parsons

**Affiliations:** Swine Teaching and Research Center, Department of Clinical Studies—New Bolton Center, School of Veterinary Medicine—University of Pennsylvania, Philadelphia, PA, United States

**Keywords:** gilt, insemination, implantation, dry sow, group housing, mental wellbeing, psychological health, sow gestation

## Abstract

Breeder animals are an important focus in farm animal welfare assessments as they typically live the longest lives and are at the greatest risk for suffering due to their longevity. For breeding pigs, the time between the end of lactation (post-weaning) and the implantation of embryos (early gestation) is very dynamic from both a physiological and husbandry perspective. However, research to date is limited on how best to house and manage sows during this critical period of their production cycle from a welfare perspective. Previous animal-based welfare outcome measures were restricted to certain health, behavioral and physiological indicators. This systematic review used Web of Science to make in-depth comparisons among welfare-based studies that focus on sow housing during the post-weaning and early pregnancy period to identify important knowledge gaps. Only a small number of studies (*n* = 27) were found that met our systematic search criteria. Compared to stalls, group housing requires mixing of animals and always triggers more aggression and skin lesions at the time of mixing. The predominant use of health and physiological indicators constrained the animal-based welfare outcomes in these studies. Thus, what type of housing yields the best overall welfare outcome remains to be elucidated as none of the studies found explored the mental wellbeing of sows during this period. This systematic review defines a critical knowledge gap regarding the full impact of housing on the welfare of post-weaning and early gestation sows. This gap, and thus the true welfare impact of sow housing, will only be addressed by the use of novel, more holistic assessment methods that also capture the psychological state of the sow.

## Introduction

The concept of cumulative assessment of an animal's lifetime experience has been recently highlighted in lab and zoo animals, and proposes that an animal's experiences, both positive and negative, will accumulate especially for long-lived animals (1). In animal agriculture, breeding animals are therefore an important focus in the welfare assessment as these animals typically live the longest lives and are at a greater risk for chronic suffering when welfare is sub-optimal on farms. In pig production, the question of confining sows during gestation has been highly debated. The European Union (EU) Pig Directive states that sows or gilts can only be confined in stalls for up to 4 weeks after insemination and 1 week before farrowing (2). In the United States (US), gestation stalls have been questioned since the early 2000's (3), and although the majority of pregnant sows are still housed in individual stalls (4, 5), some pig producers are transitioning toward group housing for pregnant sows after insemination or implantation (6). However, depending on the details of different group housing systems, post-weaned sows often still are housed individually for 3 to 10 weeks (7).

The state of California in the US passed Proposition 12 in an effort to reduce confinement for breeding animals (8). It bans all breeding sows or gilts from being housed within a confined space that is <24 ft^2^ (around 2.23 m^2^) per head, except for the 5 days prior to farrowing and during the lactation. With cessation of lactation, breeding pigs enter a period running between weaning (post-weaning) and the implantation of embryos (early gestation) that is very dynamic from both physiological and psychological perspectives. Proposition 12 is one of the few existing legislative initiatives that covers female breeding animals during early pregnancy and directs attention to this poorly studied post-weaning phase within the sow production cycle. Furthermore, this new legislation affects not only pigs raised in California, but also any pig whose pork or whose offspring's pork are sold in California. As California accounts for nearly 15% of the pork consumption in the whole US market, Proposition 12, if enacted, promises to impact sows all cross the country (9). This legislation initiative is progressive in terms of confinement-free housing and its impact on a large number of animals (potentially over 1 million sows), but scientific research to support the policy stipulations regarding post-weaned sows is lacking.

The post-weaning period is a vulnerable period for a sow as she is going through the stress of separation from her offspring and recovering from any weight loss during lactation (10). Group housing of sows during this period has the potential to negatively impact sow welfare and subsequent reproduction. Mixing sows together can result in social stress, aggression and possible injuries (10, 11), and is the main rationale for producers to house sows in individual stalls (12). The level of aggression in commercially farmed pigs can be largely attributed to the housing environment, but the composition of the group and the pig's genetic tendency during breeding selection are also shown to be important factors (13, 14). However, sows reared in semi-natural settings are in a social environment for most of their life, and they only experience isolation around farrowing (7, 15, 16). As pigs are natural omnivores and foragers, they spend 75% of their diurnal time foraging and exploring their environment with their conspecifics (17). The role of social interactions during this post-weaning phase on farms has rarely been studied.

More is known about sow behavior and welfare during the early gestation. Spoolder et al. over a decade ago reviewed factors contributing to successful sow/gilt housing during early pregnancy (10). At the time, individual sow housing after 4 days post-insemination was to be phased out in Netherlands and the authors focused on comparisons between different housing systems and management strategies used for loose-housed sows. They did not find many studies comparing different group housing systems and even fewer that went beyond assessing its impact on reproduction. Aggression resulting from the introduction of unfamiliar sows was an important challenge identified during group housing. In addition to reproductive failure, aggression can also lead to lameness, feed access competition, and variable feed intake (10). The authors suggest ample space allowance, especially in smaller groups, availability of bedding, and a welldesigned feeding system as the most important elements in successful group housing of post-weaned sows. Our current review builds on this past review (10) by also including comparisons to individual sow housing, which is still the most common practice on commercial farms globally and needs to be included in the discussion.

Housing sows in individual stalls can protect sows from early mixing and help them restore weight during the crucial stage, but this physical confinement also can cause stress, frustration and compromise welfare (12). The negative effects of stress due to confinement does not always translate into reproductive, physiological or health outcomes and can make its assessment more difficult (18, 19). Some studies examined sows' activity budget and abnormal behaviors, such as stereotypies, to determine sows' welfare status (11, 20–24). More recent research has raised questions about the psychological wellbeing of captive animals since some of them are in close confinement for most of their lifetime (25–27). Given the longevity of sows on a commercial breeding farm where animals complete several rounds of repeated confinement during gestation and farrowing, the assessment of sow welfare could learn from behavior and welfare research on other animals in captivity. Reproductive success cannot be the sole measure of welfare as it has been shown that some captive species can be reproductively active while in a poor welfare condition (26, 28).

Simple evaluation of input or resource measures, such as space allowance, feed intake or access to other resources are not adequate either. These measures fail to detect the effects of social interactions between sows and poorly capture the welfare state of individual animals. More effective sow-based outcome measures are needed to reflect their psychological as well as physical wellbeing. Furthermore, legislation, welfare concerns, and considerations about labor management often are as important in the evolution and selection of different housing systems as actual reproductive performance (4). Thus, it is important to accurately measure and understand the welfare implications of different housing systems in order to provide best possible recommendations for both the producers and the animals.

The definition of early pregnancy in this review was set at the first 4 weeks and corresponds to the completion of embryo implantation, a mechanical stabilization of the embryos in the uterus (29). The majority of embryo loss and failed pregnancies occur prior to implantation (7, 30). However, many studies looking at the impact of housing on gestating sows commence only after 4 weeks post-insemination, at a time when the pregnancy can be readily confirmed (11). In contrast, the scope of this review covers the period of early pregnancy prior to implantation, as well as the post-weaning period that immediately precedes it. Post-weaning is a dynamic and critical period for the sows and has been largely ignored in the current welfare literature. In the US, most pig farms which group house sows during pregnancy still house sows individually both post-weaning and during the first 4 weeks of gestation (31). This is also allowed in the EU where animal welfare standards are considered higher. With the advent of Prop 12 in the US and the End of Cage Age campaign in Europe (32), this review aims to provide a timely and an in-depth comparison of the relevant literature and to provide directions for future research as well as methodological refinement for better holistic assessment of sow welfare during this dynamic time of a sow's life.

## Methods of systematic literature review

**Figure 1:**
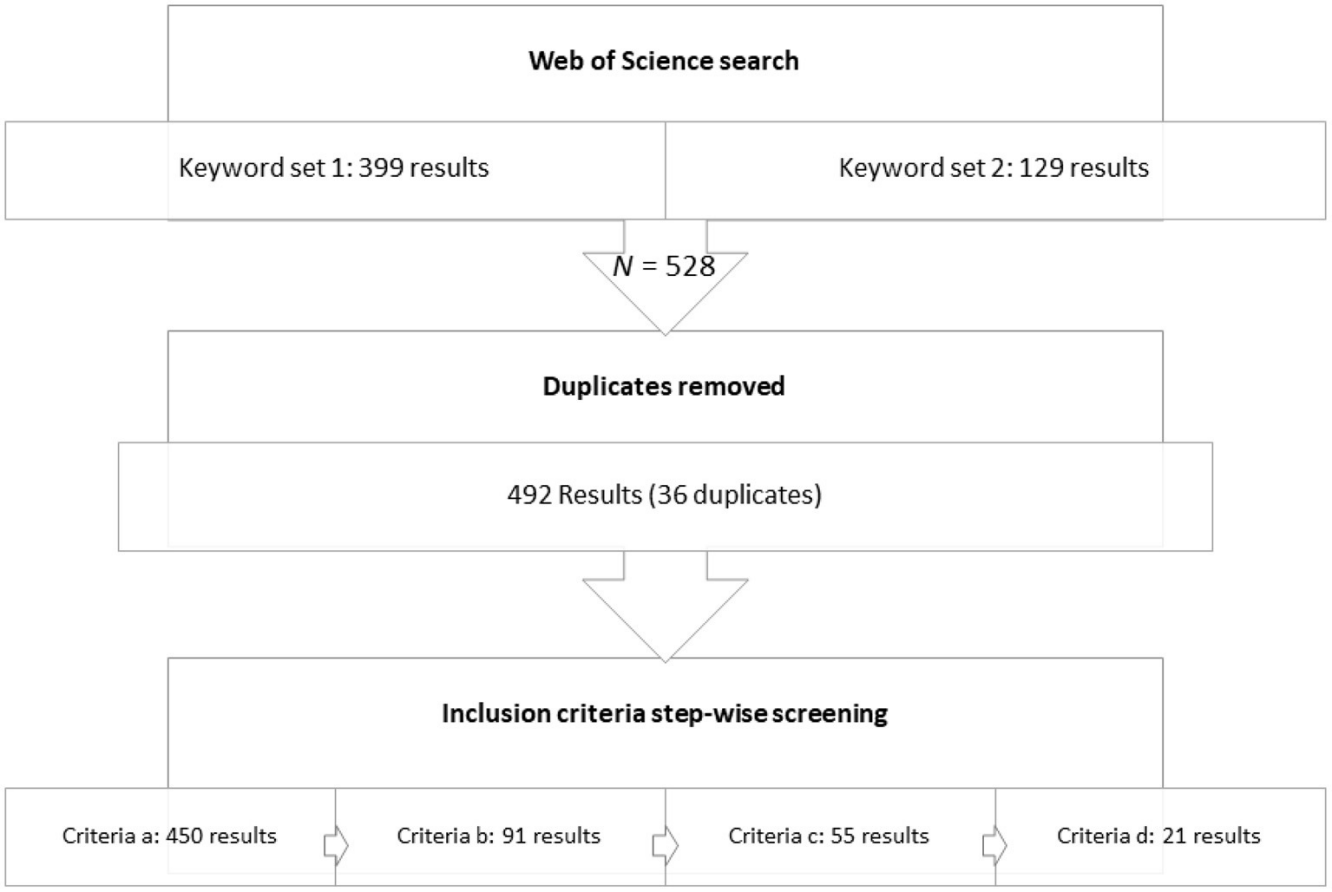
Flow chart of literature screening process (results shown from the main search).

Web of science was the database utilized to survey the current scientific literature. Two sets of keywords were employed. The first set was (sow^*^ OR gilt^*^) AND (inseminat^*^ OR implant^*^ OR bre^*^d OR service) AND housing, and the second set was (sow^*^ OR gilt^*^) AND (“early pregnan^*^” OR “early gestat^*^” OR “post-wean^*^”) AND housing. The search was conducted in “all fields” and in “all years.” Duplicates from the two sets of keyword searches were removed. The criteria of literature inclusion were: (a) it was a full scientific paper published by a peer-reviewed journal in English, (b) the discussion should be on sow/gilt and housing, (c) the focus was on the period between post-weaning to early pregnancy (first 4 weeks of pregnancy), and (d) sow-based welfare outcome measures were presented. Welfare outcome measures refer to sow-based parameters that can reflect the welfare status of the sows, e.g., behaviors, lesions, physiological measures on positive or negative emotional states. Publications that only included reproductive performances did not qualify according to this criterion. Table 1 listed the criteria based on the PICOS principles (33). In total 528 papers were obtained from the database based on the search terms (Figure 1). The search results were stepwise narrowed down based on the process described here and resulted in 21 papers that fit the criteria. In order to capture all relevant literature, another complementary search using (“weaned sow” AND housing) was conducted. An additional six papers were identified after the same screening methods described above and added to the main search results for the detailed review (*n* = 27).

**Table 1:** Inclusion criterion based on the PICOS (population, intervention, comparison, outcome and study type) framework.

**PICOS**	**Inclusion criteria**
Population	1. Housing for sows or gilts
	2. Time: during post-weaning or early pregnancy (first 4 weeks of pregnancy)
Intervention	N/A
Comparison	Comparing different housing or management strategies related to the housing aspects
Outcome	Sow-based welfare outcomes, e.g., behaviors, lesions, physiological measures on positive or negative emotional states
Study type	1. Experimental studies or reviews. 2. Peer-reviewed. 3. In English

Although sows' reproductive performance is not the focus of this review, we recognize the importance of this subject as the backdrop in the discussion of sow welfare. Therefore, the current knowledge on reproductive performance during post-weaning and early pregnancy is summarized in the next section. We then discuss the findings from the systematic review relevant to the welfare of sows in either post-weaning or post-insemination phases (early gestation) separately.

## Current knowledge on reproductive performance during post-weaning/early pregnancy

Most studies and reviews on the impact of housing on weaned sows during early pregnancy focused on their reproductive performance. Twelve previous scientific literature reviews in the past two decades have touched on the effect of housing on the early gestating sow, and seven of them centered solely on reproduction and only peripherally touched on welfare (Table 2). Some reviews covered the topic of stress and cortisol level (4, 51, 52), but also in the context of its disruption to other hormones, such as gonadotropin-releasing hormone, luteinizing hormone, progesterone or estrogen, which have influence over sow reproductive performance. Despite this large body of knowledge, the current literature remains equivocal regarding which housing types and husbandry practices are optimal for maximizing a sow's reproductive performance.

**Table 2:** List of literature (*n* = 27) which covers the topic of sow housing and welfare during post-weaning and early pregnancy, sorted chronologically within each category (experimental and review papers).

**Authors (year)**	**Title**
**Experimental papers**	
Tsuma et al. (34)	Endocrine changes during group housing of primiparous sows in early pregnancy
Durrell et al. (35)	Sow behavior and welfare in voluntary cubicle pens (small static groups) and split-yard systems (large dynamic groups)
Pedersen et al. (36)	Sexual motivation in relation to social rank in pair-housed sows
Anil et al. (37)	Effect of group size and structure on the welfare and performance of pregnant sows in pens with electronic sow feeders
Estienne et al. (38)	Reproductive traits in gilts housed individually or in groups during the first 30 days of gestation
Munsterhjelm et al. (39)	Housing during early pregnancy affects fertility and behavior of sows
Strawford et al. (40)	The effect of management strategies and parity on the behavior and physiology of gestating sows housed in an electronic sow feeding system
Elmore et al. (41)	A flooring comparison: The impact of rubber mats on the health, behavior, and welfare of group-housed sows at breeding
Hemsworth et al. (42)	Effects of group size and floor space allowance on grouped sows: Aggression, stress, skin injuries, and reproductive performance
Rault et al. (43)	Effects of group housing after weaning on sow welfare and sexual behavior
Knox et al. (44)	Effect of day of mixing gestating sows on measures of reproductive performance and animal welfare
Stevens et al. (45)	Effects of stage of gestation at mixing on aggression, injuries and stress in sows
Greenwood et al. (46)	Group and individual sow behavior is altered in early gestation by space allowance in the days immediately following grouping
Rault et al. (47)	Social interaction patterns according to stocking density and time post-mixing in group-housed gestating sows
Pierdon et al. (48)	Effect of familiarity and mixing method on gestating sow welfare and productivity in large dynamic groups
**Review papers**	
Kongsted et al. (18)	Stress and fear as possible mediators of reproduction problems in group housed sows: a review
Kemp et al. (49)	Effects of boar contact and housing conditions on estrus expression in sows
Madej et al. (50)	Stress-related effects on reproductive capacity of pigs
Einarsson et al. (51)	Stress and its influence on reproduction in pigs: a review
Spoolder et al. (10)	Group housing of sows in early pregnancy: a review of success and risk factors
Kemp et al. (4)	Reproductive Issues in Welfare-Friendly Housing Systems in Pig Husbandry: a Review
McGlone et al. (22)	Review: updated scientific evidence on the welfare of gestating sows kept in different housing systems
Einarsson et al. (19)	A 25 years experience of group-housed sows-reproduction in animal welfare-friendly systems
Verdon et al. (11)	Effects of group housing on sow welfare: A review
Peltoniemi et al. (7)	Reproduction of group-housed sows
Koketsu and Iida (30)	Sow housing associated with reproductive performance in breeding herds
Salak-Johnson (52)	Social status and housing factors affect reproductive performance of pregnant sows in groups

Most reviews reported both benefits and drawbacks on different individual and group housing systems (4, 7, 11, 18, 19). A social environment may stimulate estrus and allow more behavioral expression compared to individual confinement, but it could differ between dominant or subordinate sows due to the level of aggression and fear (4, 7, 11, 19). The presence of mature boar may facilitate sows' reproductive expression and alleviate aggression during mixing, but it also depends on the type and length of boar contact (7, 49, 53). Einarsson et al. concluded that the most sensitive times in the reproductive process are ovulation, estrus expression and implantation, when external stressors should be avoided (51). Timing of mixing, group size, social rank of the sow, the availability of bedding or other environmental enrichment, feeding and space allowance were widely recognized as the main factors to affect reproductive success in the loose housing (7, 11, 18, 19, 30, 52).

Reproductive performance is undeniably a crucial criterion in the evaluation of sow wellbeing on commercial farms; however, the lack of focus in the scientific literature on other aspects of sow welfare, especially the psychological wellbeing, deserves more attention. Therefore, this review describes more in detail the studies that used sow-based welfare measures, such as lesions, stress, behaviors and any other psychological evaluation. A list of the experimental design for each study detailed below can be found in Table 3.

**Table 3:** List of experimental studies for comparison.

**Study (year published)**		**Experimental design**					
	Breed used^*^	**Space allowance**	**Parity**	**Sample size**	**Group size**	**Feeding method**	**Treatment [exprimental duration^†^]**
**Post-weaning housing**							
Durrell et al. (35)	LW × LR	Cubicle: 4.11 m^2^/sow ESF: 1.53/1.88–3.4 m^2^/sow	Multiparous	64	Cubicle: 4 ESF: around 33	Individual stall (open) or ESF	Cubicles (static) vs. split yard ESF (dynamic) [D0–wk5]
Pedersen et al. (36)	Danish LR × YS	Pair pen: 6 m^2^/sow Individual: 6 m^2^ Solid floor with 2 kg cut straw/day	Mixed	20 gilts/19 sows	2	Feeding trough	Pair housing or individual pen [D3–D6]
Munsterhjelm et al. (39)	YS or YS × Finnish LR	Stall: 1.44 m^2^ Group: 5.1 m^2^/sow (Deep litter pen)	Multiparous 2–4 parities	12 reps × 40 sows = 480	20	Individual stall (open, drop feed)	Stall vs. group [D0–D28]
Elmore et al. (41)	LR × YS	Feeding stall: 1.06 m^2^ solid floor Pen: 5.4 m^2^ Slatted floor	Multiparous 2–11 parities	128	4	Individual stall (open)	Sow body size small or big × mat or no mat in feeding stall [D0–D10]
Rault et al. (43)	LR × LW	Stall: 2.2^*^0.6 m Group: 4.4 m^2^/sow	Mixed	360 (3 reps)	10	Individual stall (only 1^st^ feed locked in)	Stall (D6 mixed) vs. group (D0 mixed) [D0–D7]
Pierdon and Parsons (48)	PIC	Group: 1.86 m^2^/sow Stall: unknown	Multiparous	224	8	Individual stall (closed)	Familiarity (mixing post-weaning) × method of introducing into ESF pen [D0–D20]
**Post-insemination or post-mating housing**							
Tsuma et al. (34)	Swedish YS × Swedish LR	Pen: 9 m^2^ Solid floor	Primiparous	20	3	Feeding trough	Individual pen vs. group pen (rank) [D11–D17]
Estienne et al. (38)	YS × LR	Pen: 1.76 m^2^/sow Stall: 1.2 m^2^ Partly slatted floor	Primiparous	56	3	Floor feeding	Stall vs. group [D8^‣^-D30]
Hemsworth et al. (42)	LR × LW	1.4/1.8/2.0/2.2/2.4/3 m^2^/sow Partly slatted floor	Multiparous	3,120	10 or 30 or 80	Drop feeder (on floor)	Group size × space allowance [D4-10–D54]
Knox et al. (44)	PIC C-22 and C-29	1.74 m^2^/sow Fully-slatted floor	Multiparous	1,436	58	ESF	Stall vs. mixing timing (D3/D14/D35) [D3–D113]
Stevens et al. (45)	Unknown	2.3 m^2^/sow Deep litter	Multiparous	800	85	Moved to feed in individual stall	Mixing timing (D7/D42) [D7–D98]
Greenwood et al. (46)	LW × LR	Low: 2 m^2^/sow Med: 4 m^2^/sow High: 6 m^2^/sow	Multiparous	132	6	Floor feeding	Space allowance × hierarchy [D10–D14]
Rault (47)	LR × LW	High: 1.45 m^2^/sow, Moderate: 2 m^2^/sow Low: 2.9 m^2^/sow Parlty slatted floor	Multiparous	150	20 or 14 or 10	Drop feeder (on floor)	Stocking density when mixing [D4-8–D29]
**Other management strategies during post-insemination or post-mating**							
Anil et al. (37)	YS × LR	Dynamic: around 1.72 m^2^/sow Twice-mixed: 1.56–3.44 m^2^/sow Static: around 1.59 m^2^/sow	Multiparous	310	Dynamic: around 100 Twice-mixed: 22 (50) & 29 (59) Static: 24–31	ESF	Group size & structure [D10–D34]
Strawford et al. (40)	PIC	Static: 2.1 m^2^/sow + 1 ESF Dynamic: 2.1 m^2^/sow + 3 ESF Partly slatted floor	Mixed	293	Static: 34–41 Dynamic: around 105	ESF	Mixing timing × parity × familiarity of sows × static or dynamic group

† The duration here only indicates when the majority of the welfare measures were taken, and some studies continued for longer to record reproductive performance. For studies using multiparous sows, the day (“D”) always indicated the number of days post-weaning unless otherwise specified. If the days before oestrus expression were not specified, 3 days were added for post-insemination studies.

* LW, Large White; LR, Landrace; YS, Yorkshire; ESF, Electronic sow feeder.

# This study utilized all gilts and their oestrus cycles were synchronized using orally dosed progesterone and intramuscular injection of gonadotropin (with an interval of 2 days). The authors reported all gilts were inseminated within 6 days of gonadotropin injection and therefore an estimation effect of equal to 8 days post-weaning was given.

## Housing during the post-weaning period

A small number of studies have examined the welfare of sows directly weaned into pens with other sows. Durrell et al. compared weaned sows introduced into small cubicle pens (4 sows in a stable group) with free access feeding stalls to split electronic sow feeder (ESF) pens (i.e., the entrance and exit of the ESF are split into two areas without direct walkthrough access) around 33 sows in a dynamic group) (35). Sows in small cubicle pens were less active in general while sows in split ESF pens had more social interactions and agonistic encounters. Skin lesion scores were higher for sows in ESF pens compared to small cubicle pens, and they were elevated on day 1 and 4 post-introduction but by 5 weeks had returned to pre-introduction levels (35). The different activity level between the different housing systems could be due to differences in the total area of the pen or the feeding regime, as the sows were fed simultaneously once per day in the cubicle pens whereas sows went through the ESF one at time through the day. The increased aggression and skin lesions in the split ESF pens could be attributed to the more complicated social interactions associated with the larger group size or the different feeding methods.

Pedersen et al. investigated aggressive, fear and sexual behaviors between sows that were housed in pairs or individually 3 days before mating (36). Parity (primiparous vs. multiparous) also was considered as a proxy for previously experience in stall-housing. They recorded 80% of the agonistic behaviors in the first 8 h after pairing in pens. The subordinate sows suppressed their sexual behaviors and showed higher fear toward the boar compared to the dominant sows, with the individually housed animals being intermediate between the two types of sows in pens. Parity had minimal effect on all of the measures. This study demonstrated the possible effect of dominance ranking on sow behavior, but how the expression of sexual behavior relate to the mental welfare of sows is still unknown.

Munsterhjelm et al. compared sows housed in stalls or in groups of 20 in deep-littered pens with individual feeding stalls. They followed 12 batches of 40 sows from weaning until 5 weeks after when pregnancy was confirmed (39). Stalled-sows performed better in terms of some reproductive traits but showed more frustration-related behaviors, such as standing and sitting inactively. The restricted and barren environment of the stalls appeared to cause sows to redirect their behaviors toward their drinker and empty trough. However, these frustrations did not impact their reproductive performance and serves as an important reminder that reproductive success does not necessarily equate with good welfare (54). Moreover, group-housed sows in this study had both rich environmental and social stimulations, so it is difficult to interpret whether the frustration of stalled sows came from lack of social stimulation, restriction of movement, or a monotonous environment.

Looking at the sole effect of environmental modifications, Elmore et al. studied behavior, lesions and lameness of post-weaning sows housed in pens of four and equipped with open feeding stalls (41). They examined the impact of having rubber mats available under feeding stalls. Only resting behaviors were impacted by the presence of rubber mats in stalls as sows spent more time lying in the stalls, preferred to lie laterally, and made more posture changes. The mats did not influence aggressive behavior or lesion scores. Nor did sow parity influence any of the outcomes in this study. While the sows in this study demonstrated a preference for the mats, it would be interesting to investigate if additional environmental modifications, such as the provision of environmental enrichment at this stage of production, also affect sow behavior and welfare.

A more recent experimental study investigating housing for post-weaned sows was published by Rault et al. (43). Sows of similar parities were either placed into individual stalls or mixed into small groups (10 sows per pen at 4.4 m^2^/sow space allowance) immediately post-weaning (D0). Following insemination, both stalled and group weaned sows were moved on D6 to new pens housing 7 sows per pen at 2.1 m^2^/sow space allowance without adding any new sows to the groups formed on D0. They observed similar levels of aggression on D7 between sows initially grouped at D0 or D6. D0 mixed sows had higher levels of cortisol on D1 perhaps not surprising given that D6 mixed sows still were housed individually. However, by D7 once both groups had been moved into new pens, no differences in cortisol levels were observed. Interestingly, D1 cortisol levels exhibited opposite weak correlations with D0 aggression delivered (negative) and skin lesions (positive). However, similar relationships were not observed on subsequent days. As such the specificity of cortisol concentration in terms of animal welfare assessment can be complicated. Often other factors such as the sampling method, the general activity level (55, 56) or in this study post-weaning physiology (43) may have a bigger impact on cortisol concentration than social stressors associated with introduction of unfamiliar sows in groups.

Pierdon and Parsons (48) compared sows either housed individually in stalls or mixed into small groups of 8 immediately post-weaning to allow familiarization within the cohort. Eight days later inseminated sows entered a larger dynamic ESF pen and were either introduced as a cohort together or individually after feeding in the ESF stations. During the initial 8 days, group housed sows had both increased quantity and severity of skin lesions compared to stall-housed sows. Lesions remained greater on D8, when these sows were introduced into the large dynamic pen and peaked on D11, whereas the stalled-housed sows had the highest quantity of lesions on D20. No difference was found in lameness and body condition score. This work demonstrates the limitations of lesion scores as a welfare indicator as they are always confounded by absence or presence of social interaction whose possible benefits are never reflected in this measure. Taken together, housings system and management strategies varied dramatically between studies described here and unfortunately make it difficult to synthesize unified conclusions about welfare of sows weaned directly into pens with other sows.

## Housing during the post-insemination or post-mating period

Some studies housed all sows in individual stalls immediately post-weaning and only started housing treatments at some point after insemination was completed. Tsuma et al. moved a subset of animals from stalls into groups of three sows per pen or an individual pen on D11 post-insemination and compared blood levels of cortisol and the response to an ACTH challenge test conducted 5 days after regrouping. Dominance status of sows was determined by a feed competition test (34). Increased cortisol concentration was apparent in group-housed sows following mixing, especially for subordinate sows due to intense fighting observed. However, the reaction to the ACTH test did not differ between individually housed sows or group-housed, regardless of their dominance ranks, suggesting a rapid social hierarchy stabilization that failed to result in prolonged psychological stress. The elevation of cortisol observed could result from immediate effects of stress following mixing or the higher activity level because of constant displacement.

Estienne et al. moved a subset of animals from stalls into groups of three gilts per pen one day after insemination and compared gilt's stereotypic behavior and physical scores for 30 days (38). The group-housed animals had more body lesions with most lesions occurring at the front part of the body. These gilts also tended to have higher lameness scores and tended to perform more vacuum chewing. On the other hand, group-housed gilts gained more weight, which the authors attributed to better thermal regulation as gilts could huddle together when the temperature was low, but could also be explained by social facilitation during feeding (57). No difference in stereotypic behaviors was found between stalled or group-housed gilts, and since no description of environmental enrichment was provided in this study, one can speculate that barren environment or feeding regime may be more important factors behind stereotypic behaviors than the housing treatments here.

Space allowance and group size are additional factors important to the welfare of loose-housed sows. Hemsworth et al. compared different combinations of space allowance (1.4/1.8/2.0/2.2/2.4/3 m^2^ per sow) and group size (10/30/80 sows per pen) for multiparous sows that were mixed within 7 days post-insemination (D1) (42). They found a linear decrease in both aggression at the drop feeder and plasma cortisol concentration on D2 as space allowance increased, but those differences disappeared by D9. Space allowance, however, did not affect total or fresh lesion counts on any days. Group size also did not have an effect on either aggression at feeder or cortisol concentration, but sows in groups of 10 consistently exhibited lower lesion scores from D9 onwards compared to sows in larger groups. This study serves as another reminder of the collinearity of aggressive behavior and cortisol level, which may be indicative of activity level rather than affective state and calls for more specific indicators of mental wellbeing to fully understand sow welfare.

Other studies also examined the effect of space allowance during mixing when sows were grouped post-insemination. One study mixed sows 4 days post-insemination and compared the space allowance of 2, 4, or 6 m^2^ per sow during the first 3 days after mixing (46). Aggression was most pronounced on the day of initial mixing, but no differences were observed between treatments. However, sows given a larger space allowance exhibited more exploratory behavior and nonaggressive sow-to-sow contacts. Four days post-mixing, space allowance for all sows was equalized at 2 m^2^ and yielded no increase in aggression. These authors also examined the impact of social hierarchy by determining by the frequencies of winning/losing displacements and post-mixing fights. Submissive sows subjected to the lower space allowance had higher lesion scores, whereas dominant and intermediate sows provided with high space allowance exhibited fewer lesions. Another similar study, mixed sows 5 days after insemination into high (1.45 m^2^/sow), moderate (2 m^2^/sow) or low (2.9 m^2^/sow) stocking density groups (47). Low stocking density groups displayed more overall interactions between sows whereas high stocking density groups exhibited more knocking and pushing from sows as initiators. Lower cortisol concentrations were measured from sows housed at a low stocking density, but no difference in progesterone concentration was observed. These two studies demonstrated the importance of going beyond aggression to record other types of social interactions between sows. However, the difficulty remains on how to account for a lack of social interactions when comparing the welfare of individually vs. socially housed animals.

The duration of time that a sow spends in a stall post-insemination is another variable that could impact sow welfare. Knox et al. examined sows that were individually housed in stalls from weaning and left in a stall or grouped between 3 to 7 days (D3), 13 to 17 days (D14) or after 35 days (D35) post-insemination (44). Differences in sow behavior, body lesions, leg health and serum cortisol were examined across gestation, but here we will focus on early gestation findings. D14 Sows had fewer fights compared to D3 or D35 sows. Lameness and body condition score increased with increasing time in a stall post-insemination whereas head and body lesion decreased. However, lameness and lesions scores were all greater for animals in all three treatments that involved mixing compared to the sows that remained in stalls. Serum cortisol concentration was obtained before sows were assigned into treatment groups (baseline) and again on 3 and 9 days afterwards. D35 sows had the highest increase in cortisol concentration compared to all other groups. In a similar study, Stevens et al. also housed post-weaned sows in stall for 7 days until insemination was completed (45). Behavior and lesions of sows mixed immediately after service (D0) or 35 days later (D35) were compared. D0 sows displayed slightly more aggression and had higher cortisol levels on the day of mixing, but no difference in fresh lesions was observed 7 days later. However, D0 sows did have more older body lesions 7 days after mixing, suggesting lingering sporadic fighting between D0 sows after mixing compared to D35 sows. One challenge with interpreting the impact of stalling post-breeding sows in both studies is that the treatments (duration in a stall before mixing) and post-mixing observation periods are all confound by different stages of gestation.

## Management strategies during the post-insemination and post-mating period

Beside physical housing environment, some studies investigated a mix of different management strategies such as using different group structures, parities of sows or mixing strategies. Anil et al. compared behavior, lesion scores and cortisol levels between sows that were mixed either once to create a static group 5 days post-insemination, mixed-twice where sows were grouped at 5 days post-insemination and then a second cohort of sows was added 14 days later, or mixed into a dynamic group where sows were added every 14 days for the duration of the study (37). Sows housed in dynamic groups had the highest skin injury scores both on the day after mixing and two weeks later. No difference in salivary cortisol concentration was found, nor were there differences in total aggressive encounters or stereotypic behaviors. Non-agonistic social interactions were lower in the dynamic group, which could reflect less social coherence due to repeated inclusion of new animals. One challenge with studying sow housing practices on large groups of sows, as commonly used for electronic sow feeding, is that they are often logistically difficult and some compromise in experimental design may be necessary. Here, the authors were not able to completely control for space allowance or group size when manipulating the constitution of the group (e.g., static, dynamic, or twice-mixed). Furthermore, this study employed a space allowance of 1.4 m^2^, significantly less than the European Union standard of 2.0 m^2^ for groups of this size (2).

Strawford et al. used a split-plot design to study the effect of parity, mixing pre- or post-implantation, the familiarity between the sows and whether the group structure was static or dynamic on sow behavior, injuries and salivary cortisol (40). Sows or gilts were kept in individual stalls for 12 days after weaning to complete the artificial insemination and then assigned to treatments. Pigs in the post-implantation mixing treatment were kept in stalls for a further 5 weeks. Parity had the strongest effect on sow aggression with older sows (parity 4 or older) having more aggressive encounters and of longer duration. Older sows also entered the ESF earlier and laid against the wall more often than younger ones. Sows of intermediate parties (parity 2–3) had lower cortisol concentration than the older and younger sows. Mixing sows during the pre-implantation period resulted in more aggression initiated at the feeder entrance but an overall lower cortisol concentration. Group structure and the familiarity between sows only had minimal effect. No difference in injury score was found between any treatments. It can be hard to generalize conclusions from these ESF-based experiments given the complexity of these systems that often result in several factors differing between studies. These factors include pen design (square or rectangular), feeder design (where ESF tag is read to allow entry, exit strategies), flooring (slatted, partially slatted, solid, bedded), feeding protocol (daily reset time, duration of time sow allowed in feeder, time interval between feed drops) as well as experience of the barn staff with different housing/feeding systems and the quality of human-animal interactions on the farm.

## Conclusions and future directions

Our systematic review found relatively few studies on the effects of housing post-weaning and during early pregnancy that address its impact on sow welfare. The majority of the relevant literature has focused on reproductive performance. Those papers examining welfare found that group housing after weaning usually generated more agonistic interactions (35, 39, 45) and elevated cortisol concentration (34, 43, 45) compared to individual housing, especially when feeding method generates competition, such as floor feeding, an unprotected feeding stall, or queuing in front of ESF stations. Body lesions also were more prevalent due to mixing (35, 37, 38, 43–45), but some studies showed increased space allowance during regrouping can reduce aggression and the subsequent lesions (42, 47). Other studies found no difference in terms of aggression or skin lesions between different group structures (static or dynamic) (37, 40) and a limited number of studies addressing the timing of mixing did not report consistent differences (40, 43–45). Social rank and parity had some influence on agonistic interactions and consequentially the severity of lesions (34, 36, 40, 46). The main effects from the studies described above are summarized in Table 4.

**Table 4:** Summary of the main effects of the welfare outcome measures in the experimental studies reviewed.

**Study (year published)**	**Experimental design**	**Aggression**	**Stereotypies**	**Skin lesion**	**Cortisol**	**Other**
**Post-weaning housing**						
Durrell et al. (35)	Cubicles (static) vs. split yard ESF (dynamic)	ESF +	NA	ESF ++	NA	NA
Pedersen et al. (36)	Pair housing or individual pen	NA	NA	NA	NA	Subordinates +++ fear response during boar presence
Munsterhjelm et al. (39)	Stall vs. group	Group +++	NA	NA	NA	NA
Elmore et al. (41)	Sow body size small or big × mat or no mat in stall	NS	NA	Mat on D10 -	NA	Mat: rested in stall + Lameness: NS
Rault et al. (43)	Stall (D6 mixed) vs. group (D0 mixed)	NS on D7 (when stalled sows were first mixed)	NA	Postmixing lesions always +++ (no interaction with treatment reported)	Group +++ (D1)	Gait score NS
Pierdon and Parsons (48)	Familiarity (mixing post-weaning) × method of introducing into ESF pen	NA	NA	D7/8 PW mixed +++	NA	Lameness: NS BCS: NS
**Post-insemination or post-mating housing**						
Tsuma et al. (34)	Individual vs. group pen	NA	NA	NA	Group + (D1)/NS for ACTH challenge	Corticosteroid-Binding Globulin: NS Progesterone: NS
Estienne et al. (38)	Stall vs. group	NA	Vacuum chewing: Group (+)	Group ++	Stall (+)	Lameness: Group (+)
Hemsworth et al. (42)	Group size × space allowance	More space on D2 - Group size NS	NA	Group of 10 - - - (D9-51)	More space on D2 - - - Group size NS	NA
Knox et al. (44)	Stall vs. mixing timing (D3/D14/D35 post-insemination)	D14 - - -	NA	D3 + > D14 + > D35 (head)	D35 + (Serum)	Lameness: D35 + > D14 + > Stall BCS: Stall, D3, D14 +++ (increase over time)
Stevens et al. (45)	Mixing timing (D0/D35 post-insemination)	D0 +	NA	D0 ++ (old lesions on D7)	D0 + (D0)	NA
Greenwood et al. (46)	Space allowance × hierarchy	Space NS Hierarchy included: Low space + (D0 & 1)	NA	Space NS Hierarchy: submissive + in Low space	Low space - -	NA
Rault (47)	Stocking density when mixing	High +	NA	NA	Low < Med - - Low < High - - - Med < High (–)	
**Other management strategies during post-insemination or post-mating**						
Anil et al. (37)	Group size & structure	NS	NS	Dynamic ++	NS	NA
Strawford et al. (40)	Mixing timing × parity × familiarity of sows × static or dynamic group	Timing NS, Older sow +, Familiar (+), Static (+) Feeder aggression: pre-implant +	NA	Younger sow (+), Familiar (+)	Post-implant +++	Younger sows ate later +++, Post-implant ate later + Post-implant lied on slats ++ Familiar lied against wall +

^*^+++/- - -: positive/negative effects at P < 0.001; ++/- -: positive/negative effects at P < 0.01; +/–: positive/negative effects at P < 0.05; (+)/(–): positive/negative tendency at P < 0.1; NS, not statistically significant; NA, measure not available; ESF, Electronic Sow Feeder; PW, post-weaning.

Several challenges inherent in the study of sow housing and welfare were identified in this review. The implementation of group-housed sows in pens is extremely variable. Replication is rare and often precludes meaningful comparisons between studies. This work also becomes more difficult as group size increases due to the increased complexity of the husbandry practices. With the greater complexity also comes more possible confounders, such as pen and feeder design, flooring and feeding protocol. For example, behavioral time-budgets can be affected by the size of the available area and feeding regime, whilst agonistic interactions and lesion scores are affected by group size, group structure and social status. Measures like behavioral observation and lesion scoring have the potential for their outcomes and interpretation to be influenced by differences in sampling methods (see Supplementary material). Thus, future research on sow housing and welfare needs to carefully consider potential confounds and standardize sampling methods in order to facilitate comparison between studies as well as allow for meta-analysis across a large number of studies and the accumulation of knowledge necessary for identifying best practice.

The studies reviewed here employed a variety of different welfare measures. The most common methods used to assess sow welfare are behavioral budget, agonistic interactions, lesion scores and cortisol concentration (Table 4), followed by lameness and body condition scoring. However, these different measures all have their own inherent limitations. In particular, specificity of many common welfare metrics could be improved. Perhaps cortisol is the most problematic as it reflects the activation of the hypothalamus-pituitary-adrenal (HPA) axis as part of a short-term stress response. Several other physiological functions, including a natural diurnal rhythm and the hormonal changes that post-weaned sows are going through, can also influence cortisol levels, making this metric challenging to interpret when differences are observed across different housing systems. The emotional valence associated with these cortisol changes also are usually not determined or assumed to be negative. Thus, how these physiological changes impact the animal's affective state can be hard to discern.

The context of the comparison is equally important, as it can be difficult to find a method that is meaningful across all housing treatments. For instance, skin lesions will always be more severe in group-housed sows post-mixing when compared to those housed individually as the individually housing precludes sows from engaging in aggressive encounters. It is therefore imperative that the experimental design accounts for such limitations and includes a valid control treatment group to ensure a more meaningful comparison.

One additional element largely missing in the current literature is the effect of environmental enrichment post-weaning and during early gestation. While some studies reported using bedding materials (39, 45) or some quantity of straw (36), the majority of the studies were conducted in barren environment. It has been suggested that environmental enrichment can have an effect on reducing aggression in sows during mixing (20, 58). In terms of non-bedding type of enrichment, a study found that post-insemination group-housed sows interacted more with hanging ropes than a hanging rubber toy or a pine post in a fixed dispenser, although without an effect on post-mixing lesion scores (59). For stall-housed sows, enrichment promises to alleviate the frustration due to confinement (60) and deserves further investigation.

Finally, current measures largely focus on the animal's physical health and are best at identifying negative welfare states. These approaches rarely address the psychological experience of the animals and could not capture positive affective states. This asymmetry is responsible for a critical knowledge gap in our understanding of sow welfare in general, but as identified here for post-weaned and early gestation sows. Cognitive tasks (61–63) promise insight into both positive states and the animal's mental wellbeing but may not be practical for on-farm application, especially if the brevity of the critical period under investigation precludes training. Kongsted suggested there are simple behavioral tests that could assess sows' fear response on farm, such as human approach test (or its modifications) and could render similarly comparable results as the standard fear tests (18). Qualitative Behavior Assessment is proposed as a quantifiable tool to evaluate animals' emotional state and more commonly used in assessing welfare for different farm animal species (64–66). Increased consideration of the animal's psychological experience promises to reshuffle how different attributes of sow housing may be prioritized including environmental enrichment and social interactions.

Housing is more than a physical environment as it shapes a greater social dimension. The consequences of these different contributors to the sow's experience should be studied in parallel to understand fully the impact of housing on sow welfare. For example, one study found gilts prefer shorter confinement (30 min) in feeding stalls compared to a longer confinement (240 min) (67). The authors needed to refine the test to make the space in stalls more uncomfortable for the animals in order to see an effect after the 4-h confinement period. While the confinement period was relatively brief compared to what post-weaning sows normally encounter, the use of a preference test promises insight into the animal's experience. Another more recent study looking at motivation found that sows and gilts were both more willing to work for access to feed rather than additional space to exercise, but this motivation was stronger in sows that were housed in stalls previously compared to stall-naïve gilts (68). These examples demonstrate a new mindset for the study of different housing conditions that pursues the “sows' perspective.” The ultimate question is how to compare the experience of being confined in stalls, hence the restriction of movement, social interaction and boredom, with the negative consequence of aggression, lesions, elevated emotions during mixing and competition for resources. We conclude that the full impact of housing on the welfare of post-weaning and early gestation sows can only be successfully assessed using novel, more holistic methods that capture the psychological state of the sow.

## Data availability statement

The original contributions presented in the study are included in the article/Supplementary material, further inquiries can be directed to the corresponding author.

## Author contributions

J-YC and TP: conceptualization, methodology, and writing—review and editing. J-YC: investigation, writing—original draft preparation, and visualization. TP: supervision and project administration. Both authors contributed to the article and approved the submitted version.

## Funding

The authors declare that this study received funding from both the Pennsylvania Center for Poultry and Livestock Excellence and the Pennsylvania Pork Producer Council's Strategic Investment Program. The funders were not involved in the study design, collection, analysis, interpretation of data, the writing of this article or the decision to submit it for publication and therefore the research was still conducted in the absence of a conflict of interest.

## Conflict of interest

The authors declare that the research was conducted in the absence of any commercial or financial relationships that could be construed as a potential conflict of interest.

## Publisher's note

All claims expressed in this article are solely those of the authors and do not necessarily represent those of their affiliated organizations, or those of the publisher, the editors and the reviewers. Any product that may be evaluated in this article, or claim that may be made by its manufacturer, is not guaranteed or endorsed by the publisher.
