# Supplementary material for: A systematic review of the impact of housing on sow welfare during post-weaning and early pregnancy periods
Source: Front Vet Sci. 2022 Aug 23;9:903822. doi: 10.3389/fvets.2022.903822 (PMC9446151; doi:10.3389/fvets.2022.903822)
Supplement: Supplementary file 1 [file Table_1.DOCX]

Supplementary Material I

**A systematic review of the impact of housing on sow welfare during post-weaning and early pregnancy periods**

**Jen-Yun Chou, Thomas D. Parsons**

*** Correspondence:**Dr Jen-Yun Chou
jenyun.chou@gmail.com

**Supplementary material, Table 1. Methodology of behavior observation, lesion scoring and other welfare measures used and described in the experimental studies discussed in the main text**

**Table 1.** Methodology of behavior observation, lesion scoring and other welfare measures used and described in the experimental studies discussed in the main text

| **Behavior/Lesion scores** | | **Description of methods** | | **Source** |
| --- | --- | --- | --- | --- |
| **Behavior** | |  | |  |
| Rank order test | | - D4 of grouping: small feed provided in trough, repeated 10 times, order by which sows displace each other from feeding trough. | | Tsuma et al. (1996) |
| General behavior (standing, sitting, lying, locomotion, exploration), social interactions, agonistic interactions | | - Video recording (continuous 24h) - Focal sampling, during two periods of 5 min on each observation day. - In the voluntary cubicle pens, the first of the four sows was observed at 11:00 h and 15:00 h, the second sow at 12:00 h and 16:00 h, the third sow at 13:00 h and 09:00 h the following morning and the fourth sow at 14:00 h and 10:00 h the following morning. - ESF: a sow from one group of four sows was observed on the hour, a sow from a second group of four sows at 5 min past the hour, a sow from a third group of four sows at 10 min past the hour and so on. | | Durrell et al. (2002) |
| Aggression (bite/head-to-body knock, bite/head-to-body knock and parallel/inverse parallel pressing (initiator & recipient)/body turn (submission)  Sexual proceptive behavior (and signs of fear)  Sexual receptive behavior | | - Video recording (7 am to 7 pm) - All occurerence sampling - D3 (day of grouping) & D4 - The sow displaying submissive behavior most frequently during the 2×12 h observation period was identified as the subordinate in the pair - T-maze: there for 30 min twice on day 4 before the experimental testing was begun. The concrete-floored test area consisted of a 2 m×10 m runway ending in two 1.5 m×1.5 mgoal boxes. The goal boxes were separated from the stimulus compartments by iron barfences. One stimulus compartment contained an adult sexually experienced boar. The same boar was used for all sows. The other compartment was empty. A 15 cm layer of longstemmed barley straw covered the runway whereas the goal boxes were non-bedded (see Fig. 2). The test lasted for 10 min. - Mating test with a sexually experienced boar in the home pen of the boar. | | Pedersen et al. (2003) |
| Stereotypic behavior (Repeated movements, oral activities without obvious finality, rooting, and nosing occurring on successive observations, including floor licking, bar biting, bar licking, vacuum chewing, yawning and tongue movements.) | | - Direct observation starting from the beginning of the morning feeding - D28 post-mating - 1h continuous at 2-min interval | | Estienne et al. (2006) |
| Overall inactivity, exploratory, social, agonistic, locomotory, ingestive, comfort & grooming, other | | - Direct observation - D27 from an early started feeding at 2 pm - Scan sampling at 10-min interval (for 120 mins) | | Munsterhjelm et al. (2008) |
| Aggresion (parallel and inverse parallel pressing, head-to-head and head-to-body knocks, and levering), feeder entry order, lying pattern  Lying pattern (Location of resting) | | - Direct observation post-mixing for 4h - 21-23 focal sows from 34-41 in a group - Video recording of feeder aggression: 3 pm-6 am on D1/3/28/63 post-mixing - Video: 3-7 pm/7-11 pm/11 pm-6 am - Week 1/3/8 after regrouping - 3 consecutive days at 9 am - Standing or lying on solid floor against wall/solid floor centre/slatted floor | | Strawford et al. (2008) |
| Time budget: pen location, aggressive, drinking, feeding, manipulating mat, standing, resting, other, posture (lying sternally/laterally, kneeling, sitting, upright, other, backwards) | | - Video recording (time-lapse, 48h) - Post-breeding (D8/9 post-weaning) - Instantaneous sampling every 10 min | | Elmore et al. (2010) |
| Aggressive behavior at feeding (slashes, butts, pushes, and bites) | | - Video recording (continuous) - D2 & D8 (6 am-5 pm) - Continuous observations on bouts of aggressive behavior in the 30min following each feed drop (4 feeding/day) on D2 and D8 - Bout criterion interval of 5s - Only aggressive interactions in which the head of the sow (defined as extending from the snout to the ears) displaying the aggressive behaviorr was clearly visible were recorded - Instantaneous scans at 30s intervals during each 5-min block of footage were used to count the number of sows in each scan, providing an estimate of the average number of sows in the field of view during each 5-min block of the observation period. - Frequency of aggression per sow in field of view | | Hemsworth et al. (2013) |
| Fighting events (aggressive/submissive behaviors) | | - Video recording (continuous 24h) - Sows facing each other, side-by-side alignment, heads up with biting and body movement. Biting targets included face, ear, neck, body, and rump. - Aggressive encounters were considered terminated when one sow retreated. Interference from another pig was considered an additional fighting event - Pushes (given with head or shoulder) were also considered agonistic behaviors. | | Knox et al. (2014) |
| Aggression: parallel prssing (or inverse), head to body knocking, head to head knocking, biting | | - Video recording (continuous) and Go-Pro camera on feeder line - Group sows D0 & D1 (initiator/receiver): 3h post-mixing and 3h post-feeding (1^st^ feed) - Bout criterion 5s - Both groups on D7 (initiator/receiver): first 30 mins after 1^st^ feeding post-introduction - Aggression index: the ratio of aggression delivered to the total number of aggressive interactions (i.e., aggression delivered/(aggression delivered + aggression received)), varying from 0 to 1. - “Dominant” if they delivered more aggression than they received (aggression index > 0.5), “subdominant” if they received more aggression than they delivered (aggression index > 0.05 and ≤0.5), and “submissive” if they delivered very little or no aggression (aggression index ≤ 0.05) | | Rault et al. (2014) |
| Aggressive behaviors (slashes, butts, pushes and bites) | | - Direct observation - D0 (mixing) and D7 (7days after mixing). - 10-min habituation of two observers. - Bout criterion: 5s - 18 focal sows (9/observer) - Each focal sow observed for 2 min every 20 min from 9–12h (8 times) on each observation day. - Both frequency and duration. No individual ID; group level analysis. | | Stevens et al. (2015) |
| General activities (eating, drinking, standing, lying, exploring floor or pen work)/ social behaviors (displacements and fighting, knocks, bites, lunges, fleeing, mounting, and nonaggressive sow–sow contact) | | - Video recording, 7am (30 mins before feeding ) - 1pm - Continuous sampling - The total frequency of a behavior per sow, the average duration of a behavior event, the percentage of total time spent exhibiting a behavior - Hierarchy ranking: the number of successful displacements for each sow over all 4 d and the number of fights won and lost on the day of mixing. Displacements and fights were calculated as an overall or “global” rank and not based on individual resource rank, such as displacements around food, water, or space. For fights (F) and displacements (D), the sows were separated into 3 groups and hierarchy was analyzed with both displacements and fights combined, allowing a possibl 9 hierarchy groups. The ranking subgroups were: 1D or 1F sows were involved in no displacements or fights; 2D or 2F sows lost more than they won; and 3D or and 3F sows won more than they lost. For example, 1D1F sows were involved in no fights on d 0 and no displacements on d 0 to d4. | | Greenwood et al. (2016) |
| Agonistic behavior (approach, ano-genital nosing, nose to body, nose to head,threat, attempt to bite, bite to head, bite to body, head to headknock, head to body knock, parallel pressing, head to headpush, head to body push, head over body, chase, pause (<5 s),no reaction, retreat, or switch (a third pig gets involved) | | - Video recording (3 overhead cameras watched simultanrously) - D2 (day post-mixing) and D9 after 1^st^ feeding - Distinguish between initiator and receiver - Pause for 5s considered a new bout - Within eachinteraction, the series of behaviors displayed by the initiatorand the receiver was recorded in a sequential manner. | | Rault (2017) |
| **Lesion scores** | |  | |  |
| **Timing of scoring** | **Regions scored** | | **Scale & scoring system** | **Source** |
| D1 before introduction, D2,5,36 | Head, neck, shoulder, middle, hip, rear, front leg, back leg, front back, middle back and rear back | | Each site was scored separately, according to the percentage of the site containing lesions and the severity of the lesions on this site on a scale from 1 (mild) to 5 (severe) on the proportion of the whole body each site occupied, along with the percentage score and severity score for each site, was used to calculate whole body percentage and mean severity scores for each sow. These whole body percentage and severity scores for each sow were then multiplied together to provide a single skin lesion score. | Durrell et al. (2002) |
| D-1/1/14 of mixing | Ear (right and left), snout, face, forehead, shoulder (right and left sides), forelimb (right and left), neck (right and left sides), thorax (right and left sides), flank (right and left sides), top of the back (dorsum), udder, hind quarters-croup (right and left sides) and hind limb (right and left), tail, and vulva | | The frequency and severity of wounds in different body locations were scored (0 - none; 1 - mild; 2 - obvious; 3 - severe) and the scores added together to yield a total injury score. (from Anil et al. 2003) | Anil et al. (2005) |
| D0/1/3/7/14/28 | 6 regions: head, face, ears/neck, shoulders/middle body (excluding udder)/udder (ventral middle body)/rump, tail, anus, vulva/legs, feet | | 0: no blemishes or lesions  1: some reddening or mild abrasion or mild callus  2: < 10 scratches or areas of major redness  3: < 5 cuts or small wounds  4: ≥ 10 scratches, a moderate wound, some swelling, or all three  5: ≥ 5 cuts or small wounds, a severe wound, or severe swelling  Based on Arey (1999) and Harris et al. (2001) | Estienne et al. (2006) |
| D0/3/28/63, entry of farrowing, 2 week in farrowing | Scratches – head, ears, shoulders/ flanks, abdomen, udder, thighs, limbs  Other injuries - head, ears, shoulders, abdomen, thighs, limbs, udder, tail, vulva | | Scratches - Each region was scored - 0: no scratches, 1: 1-3 scratches, 2: 4-6 scratches, 3: >=7 scratches. Both fresh & old.  Other injuries - the prevalence and severity of swellings, cuts and abscesses. 0: no injuries, 1: 1-3 injuries, 2: 4-6 injuries, 3: >=7 injuries.  Total injury score: scratches + other injuries | Strawford et al. (2008) |
| D0/10 | Head, neck, shoulders /mid-body, udder/rump, tail, vulva  Hoof, toes/dew claws/lower leg/upper leg | | Mild: No blemishes/Callus or redness/< 10 scratches  Moderate < 5 cuts/Mild wound or abscess/> 10 scratches  Severe > 5 large cuts/Severe wound/Multiple abscesses  Mild: No blemishes/Alopecia or callus, redness  Moderate Mild wound/Mild swelling/Abcess  Severe Severe wound/Severe swelling/Multiple abscesses  Adapted from Arey (1999) and Boyle et al. (2000) | Elmore et al. (2010) |
| D2/9/23/51 | Each side of the sow’s body was divided into 21 areas | | 20 selected sows, the first sow sighted from the central and peripheral areas of the pens. Fresh injuries (scratches, abrasions, cuts, and abscesses), or partially healed or old injuries. From Karlen et al. (2007). | Hemsworth et al. (2013) |
|  |  | |  |  |
| D3/6/9/12 after movement (every 2 week thereafter until farrowing) | Head (and neck), body (including the shoulder, back, side, rear, and udder), leg, and vulva | | Each sow was assessed for the presence or absence of new or old lesions along with the severity of the wound based on a modification of the classification used by Salak-Johnson et al. (2007). Lesion scores were classified as none (0 = no lesions), low (1 = few lesions; moderate wounds displaying scabbing over scratch), moderate (2 = numerous wounds; scratch showing red color), or high (3 = abundant lesions; bleeding evident on wounds). Vulva lesions were categorized as none (0 = no lesions), moderate (1 = scabbing or abrasion; red in color), or high (2 = laceration and bleeding) | Knox et al. (2014) |
| D1 (post-weaning) & D7 (post-mating mixing) | Each side of the sow’s body was divided into 21 areas | | Skin injuries were categorized into fresh injuries (scratches, abrasions, cuts, and abscesses) or partially healed or old injuries. The number and the type of skin injuries were recorded, and from these records, the numbers of fresh and total injuries (fresh and old injuries) were collated for each sow on each observation day. From Karlen et al. (2007). | Rault et al. (2014) |
| D7, 42 and 91 after mixing | Both sides of the sows (the number and type of skin injuries across the sections of the sow) | | Skin injurieswere categorized as (1) scratches, (2) abrasions, (3) cuts,and (4) abscesses and these categories were termed freshinjuries. A fifth category for (5) partially healed or oldinjuries was also used and these were termed old injuries. From Karlen et al. (2007). | Stevens et al. (2015) |
| D-1/0/1/3/4 | Two sides of the body with each 21 areas | | Lesions were classified as a scratch, abrasion on skin or crack on hoof, open cut on skin or broken hoof, old cut or scar, or abscess. All injuries, including superficial, fresh, and old, were included and all areas were summed to give a total injury count. The severity of the lesions was not scored. An individual lesion was classified as any continuous wound and, if there was a break in the length of the wound, this was classified as a separate lesion. Adapted from Karlen (2007). | Greenwood et al. (2016) |
| D0/7/8/11/15/20/34/48/62/76/90/113 | Three body regions: anterior (cranial to the caudal aspect of the shoulder), side (between the caudal shoulder and cranial hip), and posterior (from the cranial hip caudally) | | Standardized scale measuring severity and quantity of lesions. Severity: 1 - Thin (<2 mm wide) and shallow; epidermis may be broken with mild surface inflammation; 2 - Moderately deep cuts (2 to 4 mm wide); epidermal and dermal layers may be broken with moderate inflammation; 3 - Occurrence of a wide (>4 mm) or deep cuts; dermal layers broken with severe inflammation. Quantity: 1 - ≤ 5 cuts; 2 - 6 to 10 cuts; 3 - 11 to 20 cuts; 4 - 21 to 30 cuts; 5 - >31 cuts. The score recorded for each region was the score from the highest scoring, most severe, lesion. | Pierdon and Parsons (2018) |
|  |  | |  |  |
| **Other measures** | |  | |  |
| Serum cortisol | | D-1 to D5 of grouping every 3h (9am-9pm): permanent jugular catheters. On ACTH challenge days, 8am-12pm & 1-3pm every 15 mins. 10ml blood sampling. (Also CBG & Progesterone)  D2/9 or 10/51 or 52: using jugular venipuncture, sows were restrained with a snout snare. At 1pm, all done within 2 minutes.  D3/9 after movement: 3 replicates with baseline samples for cortisol collected from a random selection of sows from each treatment but before movement. Blood was collected within each treatment from sows classified by lesion scores as low, moderate, and high. Blood samples (4 mL) were collected via jugular venipuncture into Vacutainer tubes following restraint of sows using a nose snare. Sampling occurred between 0900 and 1100 h, with blood obtained within 2 min of snaring.  D30: restrained with a metal snare for collection of blood samples via jugular venipuncture.  D1/7: blood samples were collected via jugular venipuncture from 3 focal randomly chosen sows within each group around 1200 h. Blood samples were collected within 2 min of restraint by snaring. On D7, blood samples were collected from the same 3 focal sows from each pen if these remained in the group postmating.  D2/26: blood samples were collected via jugularvenipuncture within 2 min of restraint by snaring (10-mL) from three focal sows within each group at 12pm. | | Tsuma et al. (1996)  Hemsworth et al. (2013)  Knox et al. (2014)  Estienne et al. (2006)  Rault et al. (2014)  Rault (2017) |
| Saliva – cortisol | | D-3/1/2/3/28/63 of mixing at 1pm  D-1/0/1/3/4: Sampling began at 1330 h on each sample day and concluded approximately 1h later  D0/7/35/42/91: 18 focal sows | | Strawford et al. (2008)  Greenwood et al. (2016)  Stevens et al. (2015) |
| ACTH challenge | | D5 of grouping at 9am intravenous injeciton of 50 IU ACTH | | Tsuma et al. (1996) |
| Lameness score | | D30 post-mating: 0 - Even strides, caudal body sways slightly while walking, gilt able to accelerate and change direction rapidly; 1 - Abnormal stride length, movements no longer fl uent, gilt appears stiff, gilt still able to accelerate and change direction; 2 - Shortened stride, lameness detected, swagger of caudal body while walking, no hindrance in gilt agility; 3 - Shortened stride, gilt displays minimum weight-bearing on affected limb, swagger of caudal body while walking, gilt will not trot and gallop; 4 - Gilt does not place affected limb on fl oor while moving; 5 - Refuses to move  D0/D10: 0 - Stands squarely on all 4 legs; Even strides; Caudal body sways slightlywhile walking; Able to accelerate andchange direction rapidly / 1 - Stands squarely on all 4 legs; Abnormal stride length (not easily identified); Movements no longer fluent (stiff); Able to accelerate andchange direction / 2 - Uneven posture; Shortened stride; Lameness detected; No hindrance in ability / 3 - Uneven posture; Shortened stride; Minimum weight bearing on affected limb; Will still trot and gallop / 4 - Affected limb is elevated off floor; May not place affected limb on the floor while moving / 5 - Will not stand unaided; Does not move  Leg assessment included lameness and leg inflammation, and scores were defined as either yes (1) or no (0) with sows observed or not observed with lameness or leg inflammation. Leg assessment was assigned once the sow in the stall was standing, whereas sows in pens were evaluated if they were standing or were induced to stand and began to walk.  D0/7/15/62/113: 0 - Sow moves easily with little inducement. She is comfortable on all her feet; 1 - Sow moves relatively easy, but a change in gate is visualized in at least one leg. She still moves easily from site to site in the barn and is not considered lame; 1.5 - Lameness is involved in one or more limbs. The sow exhibits compensatory behaviors such as dipping her head or arching her back; 2 - There is a real reluctance to walk and bear weight on one or more legs. It is difficult to move her from place to place on the farm; 3 - Sow is not bearing weight on one or more limbs. | | Estienne et al. (2006)  Elmore et al. (2010)  Knox et al. (2014)  Pierdon and Parsons (2018) |
| Gait score | | 0 = normal gait; 1 = irregular gait indicated by a visible degree of difficulty in walking but still using all 4 legs and a swagger of caudal body while walking and shortened stride; 2 = severely lame indicated by a visible reluctance to bear weight on the affected limb; or 3 = no weight bearing on affected limb or total recumbency. | | Rault et al. (2014) |
| Body condition score | | Body condition scores were assessed using the visual-appraisal method (posterior assessment of sow) described by Coffey et al. (1999), from 1 (lowest) to 5 (greatest).  D0/7/15/62/113: Score – Condition (Detect of ribs, backbone, “H” bones, and pin bones): 1 - Emaciated (Obvious); 2 - Thin (Easily detected with palm pressure); 3 - Ideal (Barely felt with firm palm pressure); 4 - Fat (None); 5 - Overly fat (None). Adapted from the Pork Quality Assurance Plus Program (<https://lms.pork.org/Tools/View/pqa-plus/program-materials>) | | Knox et al. (2014)  Pierdon and Parsons (2018) |
